# Potential Co-exposure to Arsenic and Fluoride and Biomonitoring Equivalents for Mexican Children

**DOI:** 10.29024/aogh.913

**Published:** 2018-07-27

**Authors:** Jorge H. Limón-Pacheco, Mónica I. Jiménez-Córdova, Mariana Cárdenas-González, Ilse M. Sánchez Retana, María E. Gonsebatt, Luz M. Del Razo

**Affiliations:** 1 Departamento de Medicina Genómica y Toxicología Ambiental, Instituto de Investigaciones Biomédicas, Universidad Nacional Autónoma de México, MX; 2 Departamento de Toxicología, Centro de Investigación y de Estudios Avanzados del IPN, MX; 3 Renal Division, Brigham and Women’s Hospital, Harvard Institute of Medicine, Boston, MA, US

## Abstract

**Background:**

Mexico is included in the list of countries with concurrent arsenic and fluoride contamination in drinking water. Most of the studies have been carried out in the adult population and very few in the child population. Urinary arsenic and urinary fluoride levels have been accepted as good biomarkers of exposure dose. The Biomonitoring Equivalents (BE) values are useful tools for health assessment using human biomonitoring data in relation to the exposure guidance values, but BE information for children is limited.

**Methods:**

We conducted a systematic review of the reported levels of arsenic and fluoride in drinking water, urinary quantification of speciated arsenic (inorganic arsenic and its methylated metabolites), and urinary fluoride levels in child populations. For BE values, urinary arsenic and fluoride concentrations reported in Mexican child populations were revised discussing the influence of factors such as diet, use of dental products, sex, and metabolism.

**Results:**

Approximately 0.5 and 6 million Mexican children up to 14 years of age drink water with arsenic levels over 10 µg/L and fluoride over 1.5 mg/L, respectively. Moreover, 40% of localities with arsenic levels higher than 10 µg/L also present concurrent fluoride exposure higher than 1.5 mgF/L. BE values based in urinary arsenic of 15 µg/L and urinary fluoride of 1.2 mg/L for the environmentally exposed child population are suggested.

**Conclusions:**

An actual risk map of Mexican children exposed to high levels of arsenic, fluoride, and both arsenic and fluoride in drinking water was generated. Mexican normativity for maximum contaminant level for arsenic and fluoride in drinking water should be adjusted and enforced to preserve health. BE should be used in child populations to investigate exposure.

## Introduction

Arsenic and fluorine are elements that form organic and inorganic compounds ubiquitously present in nature. Living organisms are mainly exposed to inorganic arsenic and inorganic fluoride through food and water; thus, this study focuses on environmental exposure to inorganic arsenic and inorganic fluoride. The contamination of groundwater by arsenic and fluoride is common in arid and semi-arid regions of the world, especially in oxidized and alkaline environments of several countries, mainly from Latin America, Asia, and Africa. Over 300 million people worldwide use groundwater contaminated with arsenic or fluoride as a source of drinking water. The occurrence of arsenic or fluoride in groundwater is primarily ascribed to geogenic processes. These natural sources are usually related to the dissolution of arsenic- or fluorine-containing minerals present in rocks and soils. Mining activity, smelting operations, and burning of coal are the main anthropogenic sources of arsenic and fluoride.

Children are the most vulnerable and sensitive group; *in utero* exposure is a recognized condition of vulnerability. However, the total number of children exposed to arsenic or fluoride or concurrent exposure to both elements through drinking water in Mexico has not been clearly determined.

## Methods

### Estimation of Mexican Children Exposed to Arsenic and Fluoride

Data of water quality were obtained from the Inventario Nacional de Calidad del agua (INCA) [1] during sampling campaigns of underground wells carried out from 2005 to 2016. Localities were established according to the georeferencing data of the sampling site. Each site was verified using the national Water Geographic Information System (SIGA) [2]. Sites whose water use corresponded to public services, industrial use, or agricultural production areas located more than 300 meters away from a population were excluded. Sites where arsenic or fluoride was relevant for child exposure were considered as follows: arsenic ≥10 µg/L and fluoride ≤1.5 mg/L. The localities were considered only if the concentration of fluoride was ≥1.5 mg/L and arsenic <10 µg/L. Localities with co-exposure were included if the values of arsenic and fluoride exceeded the concentrations mentioned above. The number of individuals between 0 and 14 years of age was considered as the child population. Sites were represented using Quantum GIS [3] and maps obtained from Instituto Nacional de Estadística y Geografía (INEGI) [4]. The population exposed was estimated using the 2010 census at the INEGI for each locality enlisted [4]. Our estimation of exposed child population may be larger because the child population has increased since 2010.

### Biomonitoring Equivalents (BE)

This review was performed considering those studies that reported levels of arsenic and fluoride in drinking water, urinary quantification of speciated arsenic (inorganic arsenic and its methylated metabolites), and urinary fluoride levels in child populations. For BE values, urinary arsenic and fluoride concentrations reported in child populations were revised.

## Results

### Overview of Arsenic Exposure in Mexico and Its Health Effects

The World Health Organization (WHO) recommends 10 µg/L as the maximum level of arsenic in drinking water [5]; however, in Mexico, the guideline value is 25 µg/L (Table 1) [6].

**Table 1:** Actual Guidelines and Standards for Arsenic and Fluoride in Water, Food, Dentifrice, and Soil.

Water			

Toxicant	Guideline, Criteria (year of evaluation)	Value mg/L	Ref

Arsenic	Drinking water quality (2004)	0.01	WHO, 2011 [5]
	Drinking water quality (2000)	0.025	SSA, 2000 [6]
	MCL (2002)	0.01	EPA, 2002 [7]

Fluoride	MCL, Drinking water (2003) SMCL, Drinking water (2003)	4.0 2.0	EPA, 201 [8]
	Drinking water quality (2004)	1.5	WHO, 2011 [5]
	Recommendation, Drinking water (2015)	0.7	US SHHS, 2015 [9]
	PL, fluoride in tap drinking water (2000)	1.5	SSA, 2000 [6]
	MPL, fluoride in bottled water (2015)	0.7	SSA, 2015 [10]
	ML, fluoride in bottled natural mineral water (2015)	2.0	SSA, 2015 [10]

**Food and toothpaste**			

Toxicant	Product, Guideline (year of evaluation)	Value mg/Kg	Ref

Arsenic	Action level for inorganic arsenic in apple juice (2011)	0.01	FDA, 2013 [11]
	ML, salt, food grade (1985)	0.5	FAO/WHO, 2011 [12]
	ML, animal fats and vegetable oils (1981 and 1999).	0.1	FAO/WHO, 2011 [12]
	ML, polished rice, rice cereals (2014)	0.2	FAO/WHO, 2014 [13]
	Raw milk, pasteurized milk, sterilized milk, modified milk, fermented milk (2014)	0.1	MOPH, 2014 [14]
	Milk powders (2014)	0.5	MOPH, 2014 [14]
	Maximum Level of inorganic arsenic in infant rice-based products (2015)	0.1	US. 2015 [15]
	Action level for inorganic arsenic in infant rice cereals (2016)	0.1	FDA, 201 [16]

Fluoride	ML, infant formula (1981)	100 µg/100 kcal	FAO/WHO, 2007 [17]
	MML, dentifrice for children (1996)	850–1150	FDA, 1996 [18]
	MML, salt (2010)^a^	200–250	SSA, 2010 [19]

**Soil**			

Toxicant	Guideline, Residential Use, Dry Weight (year of evaluation)	Value mg/Kg	Ref

Arsenic	Action level for As in contaminated soil (2004)	22	SEMARNAT, 2004 [20]
	SGV (2009)	32	UKEA, 2009 [21]
	SQG (2007)	12	CCME, 2007 [22]

Fluoride	Non-Established^b^	—	—

*Abbreviations*: MCL, maximum contamination level; MAL, maximum acceptable limit; ML, maximum level; RSL, regional screening. evel; THQ, target hazard quotient; SGV, soil guideline value; SQG, soil quality guideline; SSA, secretaria de salud; PL, permissible limit; MML, minimum and maximum level. *Noncancer Child Hazard Index (HI) = 1. ^a^In regions with fluoride concentration under 0.7 mg/L in drinking water. ^b^No international or national guideline for soil are established. However, a mean concentration of 321 mg of fluoride per kilogram of soil has been reported as baseline criteria for soils [23].

**Figure 1:**
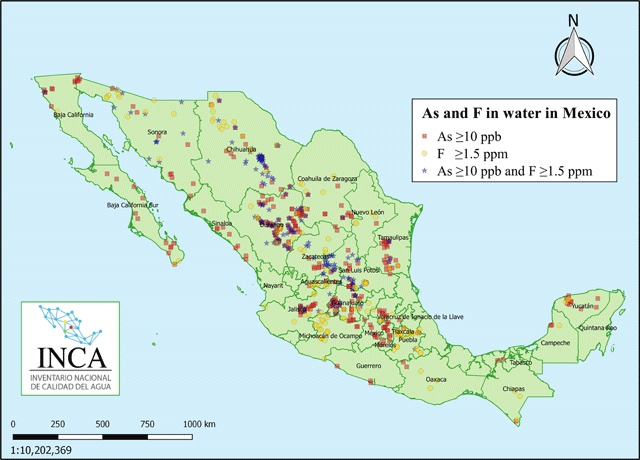
Sites with presence of arsenic, fluoride, or its co-presence in well water in Mexico. Squares: arsenic ≥10 µg/L and fluoride ≤ 1.5 mg/L. Circles: localities where fluoride was ≥1.5 mg/L and arsenic <10 µg/L. Stars: localities with co-presence of arsenic and fluoride that exceeded the levels mentioned.

There are many regions in Mexico where the natural concentration of arsenic in the groundwater depends on the arsenic content of the bedrock; here, the levels of arsenic in drinking water are higher than the recommended limit of 10 µg/L (Figure 1) and higher than Mexico’s limit 25 µg/L. We estimated that approximately 500,000 children up to 14 years of age drink water with arsenic levels over 10 µg/L, around 205,000 of them drink water with 25 µgAs/L, and approximately 17,500 children are at higher risk because their drinking water contains between 75 and 500 µgAs/L. The additional child population *in utero* was not included in this estimation.

The general population is exposed to arsenic by drinking water, eating food, or consuming soil polluted with high levels of arsenic.

### Health Effects

Arsenic accumulates mainly in the skin, causing cutaneous alteration; changes in pigmentation and hyperkeratosis are the best described and characterized toxic signs of chronic exposure to arsenic, mostly in the adult population. However, skin changes were documented in children 6 to 8 years old. Other adverse effects described in children are genetic damage, impaired learning and memory abilities, neuropathies, cardiovascular alterations, endocrine dysfunctions, and immunosuppression [24].

### Neurotoxic Effects

In San Luis Potosí, children’s verbal skills, long term memory, and linguistic abstraction were negatively associated with increased levels of arsenic in urine [25]. In the Laguna region, the most-studied area, neurotoxic effects of arsenic, such as impaired cognitive and behavioral functions, were described in a child population of approximately 600 children between 6 and 8 years of age [26,27]. In Durango state, arsenic exposure was also associated with impaired cognitive abilities [28].

### Immunotoxic Events

At the Zimapán area, children 6 to 12 years old showed impaired immune function, allergies, and altered cellular proliferation [29]. Also, there are studies documenting immunosuppression [30] and chronic inflammation biomarkers. In the Laguna region, a study including 275 children showed 58% having a restrictive spirometric pattern, while the highest exposed group showed arsenic-induced alterations in inflammatory biomarkers. The authors suggest that chronic inflammation might contribute to the development of restrictive lung diseases that could start *in utero* [31,32].

### Genotoxicity

Genetic damage is considered a biomarker of cancer risk. Arsenic is a genotoxic agent. Similar to adults, children showed genetic damage associated with arsenic exposure [33,34,35]. Epigenetic changes were observed in cord-blood cells [32,36,37,38] and in leukocytes from child populations exposed to arsenic in drinking water [39] or in historic mining areas [40].

### Cardiac and Renal Effects

At the Zimapán area, studies identified a higher risk for cardiac diseases such as atherogenesis, arterial hypertension, cardiac hypertrophy, and dysfunction [41,42]. In Villa de Reyes, San Luis Potosí, children showed early signs of kidney damage [43].

### Overview of Fluoride Exposure in Mexico and Its Health Effects

Drinking water is the major contributor to fluoride exposure. In children, this source contributes 60% to 80% of total daily fluoride intake [44]. Water fluoride regulation values are shown in Table 1. Mexico’s limits are 1.5 mg/L in tap water, 0.7 mg/L in bottled water, and 2 mg/L in natural mineral water [6,10]. In Figure 1, the presence of high natural fluoride levels in Mexico’s tap water are shown. We estimated that in Mexico approximately 6 million children up to 14 years of age drink water with fluoride levels over 1.5 mg/L and approximately 400,000 children are at higher risk because their drinking water exceeds 4.5 mg/L. In these estimations, we did not consider pregnant women for *in utero* exposure.

Fluoride toothpaste use has been one of the most common strategies to prevent dental caries in Mexico and worldwide. Studies have reported that between 49.8% to 90.9% of children 6 to 13 years old use fluoride toothpaste at least once a day [45]. Toothpaste fluoride concentrations for children in Mexico varied from nondetectable to 1153 mg/Kg, with an average of 563 ± 350 mg/Kg [46]. These large variations are due in part to the lack of an official regulation and nonmandatory normativity [47]. Children younger than 6, especially those not supervised by an adult, are at higher risk of developing dental fluorosis due to ingesting excessive dental products. Mexican regulations have established the use of a pea-sized portion of toothpaste with 550 mg/Kg of fluoride concentration under adult supervision for children from 2 to 6 years. Excessive quantities and adult toothpaste (1000 mg/Kg of fluoride) use by children under 6 was reported [48]. Moreover, an increase in dental fluorosis associated with ingesting dental products has been reported in Mexican children 11 to 12 years old [49]. It has been estimated that toothpaste can contribute at least 20% of the total fluoride exposure in children [50].

### Fluoride Enriched Supplements

Since 1940, the Pan American Health Organization has proposed fluoride food enrichment in zones with low fluoride water concentrations to prevent tooth decay. A dose of 0.05 mg/Kg per day was proposed to prevent tooth decay in children. However, evidence suggests that the predominant caries-preventive effect of fluoride is by its topical rather than systemic effect [51]. In Mexico, a salt fluoridation program to prevent tooth decay was introduced in some states in 1991. Mexican regulation establishes 250 mg/Kg of salt (Table 1) [19]. This regulation establishes no fluorinated salt distribution in zones with natural water fluoride concentrations above 0.7 mg/L to protect the population from dental fluorosis. However, 39% to 69% of children (n = 475) from areas with fluorinated salt distribution and exposure to optimal fluoride concentrations (0.7 and 1.5 mg/L) in drinking water showed dental fluorosis [52].

Besides drinking water and dental products, food can contribute up to 20% of total daily fluoride intake. The most important foods in terms of potential contribution to fluoride exposure are infant formula, commercial beverages such as juice and soft drinks, grapes and grape products, teas, and processed chicken. Infant formula and commercial beverages greatly depend on the fluoride content of the water used in their preparation [44]. Consumption of soft drinks and sweetened beverages has been reported in up to 79% of Mexican children [53]. Also, fluoride concentrations above 0.7 mg/L were reported in bottled beverages such as water, juices, nectars, and soft drinks consumed in some regions [54].

Soil could be an additional source of fluoride exposure for children. A study performed in the Laguna region reported soil fluoride concentrations between 89.75 to 926.63 mg/Kg, and the estimated percentage of bio-accessible fluoride from this source was between 2% and 46% [23]. The estimated value for incidental soil ingestion by a 20 Kg child with no pica habit is 100 mg/day. Nevertheless, fluoride intake due to soil has not been considered.

### Health Effects

Worldwide, excessive fluoride ingestion has been associated with dental and skeletal fluorosis and nonskeletal adverse effects, such as neurocognitive alterations, thyroid dysfunction, kidney injury, and cardiovascular alterations.

### Dental and Skeletal Fluorosis

Dental and skeletal fluorosis is the most common adverse effect associated with chronic fluoride exposure. Dental fluorosis cases are characterized by a hypomineralization of the enamel surface [55]. The prevalence of dental fluorosis in Mexico has been evaluated in several studies. A study review including 14 child population studies published from 1979 to 2001 observed a dental fluorosis prevalence range from 30% to 100% [56]. Another review that included 17 studies performed in Mexican communities from 10 states between 2005 and 2015 reported a prevalence range of 15.5% to 100% [57]. This high prevalence even in regions considered to have optimal or suboptimal fluoride concentrations suggests an important contribution from other sources, which should be considered in a reevaluation of the concept of optimal fluoride concentration in water. A reduction of the optimal fluoride concentration in drinking water to 0.7 mg/L has been proposed [9]. Skeletal fluorosis, which is a bone disorder characterized by an increased bone mass and radiographic density, have not been reported in Mexican children.

### Neurodevelopmental Toxicity

One of the most nonskeletal adverse effects studied in fluoride-exposed children is cognitive dysfunction. In endemic fluorosis regions, children who live in areas with high fluoride exposure reported lower IQ scores [58]. Similarly, a cross-sectional study of 132 Mexican children reported an association between fluoride exposure and reduced cognitive, verbal, and full IQ scores [28]. The main windows of susceptibility for neurodevelopmental toxicity are *in utero*, infancy, and early childhood. In a longitudinal birth cohort study, fluoride in maternal urine in the first (1.9 mg/L) and second (2.0 mg/L) trimesters was negatively associated on the mental development index in Mexican infants 3 to 15 months old, suggesting neurodevelopmental adverse effects due to fluoride exposure [59].

### Endocrine Disruption

Fluoride is considered as a possible endocrine disruptor. Studies reported a decrease in circulating thyroid hormones (Free T4 or thyroid stimulating hormone) in children exposed to high natural fluoride levels in drinking water in India (1.6 to 5.5 mg/L) and China (mean 2.36 ± 0.7 mg/L) [60,61]. In Mexico, there are no studies on fluoride exposure and thyroid function in children.

### Early Kidney Injury

The kidneys are susceptible to fluoride exposure damage. Ecological studies reported a relationship between environmental fluoride levels and chronic kidney disease of uncertain etiology [62]. However, limited information from epidemiological studies is available in part due to the lack of human early kidney injury biomarkers. In 2007, a study performed in China reported a significant increase in two early kidney injury biomarkers in children exposed to 2 mg/L of fluoride in drinking water, suggesting early injury by fluoride exposure [63]. Because evidence is limited, more studies are required to assess kidney injury due to fluoride exposure.

### Cardiovascular Diseases (CVD)

Atherosclerosis, hypertension, and cardiac dysfunction in adults were associated with fluoride exposure [64]. In children, limited information is available about the effects of early fluoride exposure. A child study performed in Turkey reported an increased electrocardiographic Q-T interval, suggesting a vulnerability for children developing arrhythmias [65]. Thus, more studies are necessary to assess the effect of fluoride in CVD development.

### Potential Co-exposure of Arsenic and Fluoride

The co-occurrence of high arsenic and fluoride levels in drinking water has been reported in many geographical regions. In Mexico, around 40% of localities with arsenic levels higher than 10 µg/L also present concurrent fluoride exposure higher than 1.5 mgF/L, especially in the central and northern regions of the country (Figure 1). Little is known about the adverse health effects from long-term exposure to arsenic and fluoride at low and high doses. The concurrent exposure may lead to a network of both synergistic and antagonistic interactions that could represent a serious health risk. Thus, the potential role of fluoride in health effects previously attributed to arsenic alone should be systematically studied.

### Biomonitoring Equivalents (BE) for Arsenic

#### Exposure Guidance Value

The current standards and guidelines of safe exposure to arsenic are summarized in Table 2. The reference dose (RfD) for arsenic is 0.0003 mg/Kg-d, the no-observed-adverse-effect and lowest-observed-adverse-effect levels were derived from this value. Additionally, it corresponds to the total daily intake (TDI) and the minimum risk level for chronic exposure.

**Table 2:** Exposure Guidelines Values for Oral Exposure to Arsenic and Fluoride.

Organization Ref	Guideline, Criteria (year of evaluation)	Study Description	Endpoint and Dose	Value	Experimental Doses (mg/Kg-day)

Arsenic Non-Cancer Endpoints					

US EPA Health Canada [66]	RfD (1993), TDI (2008)	Human cohort exposed to arsenic in drinking water	Hyperpigmentation, keratosis, and ar complications	0.0003 mg/Kg-d	NOAEL: 0.0008 LOAEL: 0.014
ATSDR [66]	MRL, chronic (2007)	Human cohort exposed to arsenic in drinking water	Dermal effects in a farming population	0.0003 mg/Kg-d	NOAEL: 0.0008
ATSDR [67]	MRL, acute (2007)	Human cohort exposed to contaminated soy sauce	Facial edema and gastrointestinal effects	0.005 mg/Kg-d	LOAEL: 0.05
US EPA [68]	Ingestion SL Child THQ = 1 (2017)*	_	_	0.39 mg/Kg	_
**Arsenic Cancer Endpoints**					

US EPA [66]	OSF (2007)	Human cohort exposed to arsenic in drinking water	Skin cancer	1.5 mg/Kg-d	_

**Fluoride Non-Cancer Endpoints**					

US EPA [69]	RfD, chronic (2010)	Cross-sectional study in children exposed to fluoride in drinking water	Severe dental fluorosis	0.08 mg/Kg-d	NOAEL: 0.08
ATSDR [70]	MRL, chronic (2003)	Cross-sectional study in adult Chinese population exposed to fluoride in drinking water	Risk of bone fractures	0.05 mg/Kg-d	NOAEL: 0.15
Health Canada [71]	TDI, chronic (2010)	Cross-sectional study in children exposed to fluoride from fluids and food	Moderate dental fluorosis	0.105 mg/Kg-d	NOAEL: 0.105
IOM [72]	UL, children under 8 years (1997)	Studies in children exposed to fluoride from dietary sources	Moderate dental fluorosis	0.1 mg/Kg-d	LOAEL: 0.1
	UL, children ≥ 8 years (1997)		Early signs of skeletal fluorosis	10 mg/day	NOAEL: 10 mg/day

*Abbreviations*: RfD, reference dose; TDI, total daily intake; MRL, minimum risk level; SL, screening level; THQ, target hazard quotient; OSF, oral slope factor; UL, upper intake; NOAEL, non-observable adverse effect level; LOAEL, low-observable adverse effect level. * Non-Cancer Child Hazard Index (HI) = 1.

Food sources, especially rice-based products, are increasingly recognized as a source of arsenic exposure for children [73]. New regulations set arsenic content in rice products for infants and young children to 0.1 mg/Kg (Table 1).

Because children engage in frequent hand-to-mouth behaviors and live and play close to the ground, they are generally more likely to have higher exposure to soil contaminants. The Canadian soil quality guideline is one of the most restrictive at 12 mg/Kg (Table 1) [22]. In Mexico, the guideline established 22 mg/Kg as the action level for arsenic contaminated soil (Table 1) [20].

#### Toxicokinetics of Arsenic in Children

Arsenic is absorbed primarily through oral ingestion or inhalation. Absorption differences between children and adults have not been reported. Absorbed arsenic binds to red blood cells, may pass through the placenta, and deposits itself in the liver, kidneys, urinary bladder, muscle, brain, bone, hair, skin, and nails. Arsenic metabolism is a process that begins with arsenate, which is converted into arsenite for its methylation into monomethylated (MAs) and dimethylated (DMAs) arsenicals, before being excreted in the urine. Arsenic urine concentrations in children are typically higher than adults exposed to the same concentrations, suggesting children accumulate less arsenic due to the activity of their metabolism, which is different from adults [74,75]. Children might have higher arsenic methylation than adults within a certain range of arsenic exposure concentrations [75]. In relation to sex, boys present higher arsenic levels in urine and less ability to methylate arsenic than girls [76].

#### Derivation of Biomonitoring Equivalents

A BE represents the concentration of a chemical or its metabolite(s) in biological specimens compared to an acceptable level of exposure based on existing exposure guidelines values (e.g., a RfD, MRLs, or TDI) [77]. A BE is derived by integrating available data on toxicokinetics with the health-based exposure guidelines to quantitatively interpret population-based biomonitoring results in a public health risk context [77]. The BE value for urinary arsenic, which is the sum of inorganic arsenic and its methylated species, is 6.4 µg/L (8.3 µg/g creatinine) [78]. This BE is derived from recent available health-based exposure guidance values (risk-specific doses for cancer endpoints) from several international agencies and the integration of controlled human dosing toxicokinetic data (urine excretion) [78]. Hence, the arsenic BE is derived for the simple exposure scenario of continuous steady-state exposure. Comparisons to this BE value should be made only with the sum of inorganic arsenic + MAs + DMAs. Hays et al indicate that biomonitoring results above the point of departure BE (BE_POD_ = 19.3 µg/L or 24.9 µg/g creatinine) should be considered as high priority for risk assessment follow-up [78].

The German reference value (RV_95_) established for children (3 to 14 years old) who did not eat fish 48 hours prior to sample collection has a similar derivation strategy, but RV_95_ is equal to 15 µg/L and is within the 95% confidence interval of the 95th percentile of the concentration of urine arsenic in a reference population sampled in the German Environmental Survey [79]. Hence, 15 μg/L represents the body burden of arsenic in a representative population from Germany and can be used only as an indicator of higher-than-usual internal exposure levels and knowing that it is linked to a specific country or region [79].

### Biomonitoring Equivalents for Fluoride

#### Exposure Guidance Values

Health-based noncancer fluoride guidance values have been reported by many agencies (Table 2). These values are based on objectionable fluorosis (moderate to severe) and skeletal fluorosis as a disease criterion. However, most of those events appear in high doses of exposure and might not be adequate for infants and younger children (3 to 6 years old) who are at highest risk due to their body mass, high ingestion of dental products, and metabolism [44]. Further, other nonskeletal adverse effects, such as neurocognitive dysfunction, can occur at low doses of exposure, which can be useful as a disease criterion [80].

Many sources of fluoride contribute to TDI. National and international environmental quality standards have been established (Table 2). In Mexico, regulations are available only for water and salt, which establish the exclusive distribution of fluorinated salt in regions with fluoride water concentrations under 0.7 mg/L [19].

#### Toxicokinetics of Fluoride in Children

Fluoride is mainly absorbed through the gastrointestinal tract. No absorption differences between adults and children have been reported. However, studies suggest that factors such as undernutrition status and low water concentration of some ions, such as Ca^+2^, Mg^+2^, and Al^+3^, can increase fluoride absorption and bioavailability [81]. Fluoride is rapidly distributed by the bloodstream to soft tissues, is readily transferred across the placenta, and accumulates mainly in mineralized tissues such as bones and teeth. Fluoride retention is 20% higher in growing children than adults, mainly due to a higher fluoride uptake in developing bones [70]. Urine is the main route of fluoride excretion. Lower urinary fluoride excretion has been reported in children, because children present the highest accumulation in calcified tissues and the highest urine flow rates [82].

#### Derivation of Biomonitoring Equivalents

Potential sources of fluoride exposure have been described above. Estimation of TDI requires their identification and exposure magnitude, frequency, and duration [83]. Urinary fluoride concentration is a biomarker of fluoride exposure, mainly due to the significant correlation with fluoride intake and the low invasiveness of sample collection [44]. However, no reference values (RV) for environmentally exposed populations have been established. Recently, Aylward et al. reported BE for fluoride [84]. These values can be calculated using data from studies that relate urinary fluoride excretion to daily fluoride intake and estimated body-weight adjusted daily urinary volume or creatinine excretion in children.

### Children Biomonitoring Reference Values for Arsenic and Fluoride

Table 3 shows biomonitoring data of urinary arsenic in children from nonendemic populations around the world. They represent general worldwide background RV of arsenic in children. In general, the 95th percentile of the sum of arsenical concentrations is <20 μg/L, both corrected or not, for urine dilution. All data are below the German Biological Tolerance Value (BAT = 50 μg/L) and the Biological Exposure Index (BEI® = 35 μg/L); both RV for adults are in occupational settings for German and US agencies, respectively. Most of the studies presented in Table 3 present levels higher than the BE (6.4 μg/L). Importantly, the average of the 50th percentile of arsenic concentrations in endemic regions of Mexico is around 80 μg/L, higher than BE, RV_95_, BEI®, or BAT (Table 4).

**Table 3:** Human Biomonitoring Data of Arsenic and Fluoride in Urine from Surveys in Children from Non-endemic/Reference Populations.

Arsenic Biomonitoring								

Country/Ref	n	Age (years)	Total As	SumAs	iAs	MAs	DMAs	Urine Dilution Adj.

Canada, Health 2014–2015 [71]	513	6–11		95th: 18 µg/L 50th: 5.5 µg/L		95th: 1.4 µg/L 50th: <LOD	95th: 14 µg/L 50th: 3.7 µg/L	None
Germany [85]	1734	3–14		95th: 14 µg/L 50th: 4.5 µg/L				None
Asaluyeh City, Iran [86]	368	6–12	C: 3.0 µg/gCr R: 2.2 µg/gCr					Urinary Creatinine
Yucatán, México [87]	36	6–9		7.5 µg/L				None
Yucatán, México [88]	107	6–9		2.9 µg/L				None
Montevideo, Uruguay [89]	327	5–9		9.9 µg/L	1.0 µg/L	0.9 µg/L	1.9 µg/L	Specific Gravity
Asturias, Gipuzkoa, Sabadell and Valencia, Spain [90]	400	4			0.3 µg/L	0.4 µg/L	3.9 µg/L	Specific Gravity
Huelva, Spain [91]	261	6–9	95th: 20.8 µg/gCr 50th: 3.4 µg/gCr					Urinary Creatinine
US, NHANES 2011–2014 [92]	397	6–12		95th: 17.8 µg/gCr 50th: 6.7 µg/gCr		95th: 1.7 µg/gCr 50th: <LOD	95th: 13.3µg/gCr 50th: 4.6µg/gCr	Urinary Creatinine
US, NHANES 2003–2008 [93]	2323	6–17	C: 8.9 µg/g Cr R: 5.5 µg/g Cr				C: 6.0 µg/gCr R: 3.6 µg/gCr	Urinary Creatinine
US, NHANES 2003–2004 [94]	290	6–11	95th: 38.2 µg/gCr 50th: 7.1 µg/gCr	95th: 14.7 µg/gCr 50th: 6.0 µg/gCr			95th: 13.9µg/gCr 50th: 4.0µg/gCr	Urinary Creatinine
**Fluoride Biomonitoring**								

**Country/Ref**		**n**	**Age/years**	**Source of Exposure**	**F Drinking water**	**F in urine**	**Urine Dilution Adj**	

Trinidad and Tobago 2001 [95]		500	6–14	NR	NR	Mean: 0.57 mg/L	None	
Iztapalapa, Mexico City [96]		205	4–5 11–12	Various	0.27 mg/L	Mean: 0.84 mg/L Mean: 0.58 mg/L	None	
China 2008–2009 [97]		26931	8–12	Drinking Water	0.54 mg/L	50th: 0.90 mg/L 75th: 1.51 mg/L	None	
Health Canada 2014–2015 [71]		533	6–11	Various	NR	95th: 1.6 mg/L 50th: 0.47 mg/L	None	
United Kingdom 2002–2014 [98]		158	1.5–7	Various	NR	Mean: 1.21 ± 0.6 mg/gCr	Urinary Creatinine	

*Abbreviations*: SumAs, sum of arsenic; C, case; R, reference; F, fluoride; NR, non-reported *Notes*: Total As includes all As species, organic and inorganic, whereas SumAs are: As(III) + As(V) + MAs + DMAs.

**Table 4:** Summary of Recent Human Biomonitoring Data and Health Effects Associated with Arsenic and Fluoride Exposure in Children from Endemic Regions in Mexico.

Arsenic Biomonitoring								

Region/Ref	n	Age (years)	SumAs	iAs	MAs	DMAs	Urine Dilution Adj.	Health Effect(s)

Torreón, Coahuila [76]	591	6–8	52.1 µg/L	7.3 µg/L	6.4 µg/L	38.2 µg/L	None	Reducing As Methylation Capacity
Torreón, Coahuila [27]	526	6–7	55.2 µg/L	7.5 µg/L	6.7 µg/L	39.3 µg/L	None	Poor Behavior
Torreón, Coahuila [26]	591	6–8	58.1 µg/L	8.7 µg/L	7.7 µg/L	41.7 µg/L	None	Cognitive Deficits
Ciudad Juárez, Chihuahua [119]	135	6–12	95th: 48.9 µg/gCr 50th: 17.6 µg/gCr				Urinary Creatinine	Biomonitoring
La Laguna, Durango [32]	358	6–12	H: 294.0 µg/L M: 143.7 µg/L L: 84.9 µg/L	H: 51.6 µg/L M: 23.9 µg/L L: 18.4 µg/L	H: 40.7 µg/L M: 19.6 µg/L L: 10.8 µg/L	H: 189.9 µg/L M: 96.2 µg/L L: 53.3 µg/L	None	Decreased Lung Function
Taxco, Guerrero [99]	50	6–10	16.5 µg/L				None	Biomonitoring
Zimapán, Hidalgo [41]	195	3–14	59.1 µg/L	5.4 µg/L	5.4 µg/L	46.7 µg/L	None	Early Cardiovascular Effects
Zimapán, Hidalgo [100]	87	6–10	194.6 µg/gCr	20.4 µg/gCr	30.1 µg/gCr	144.1 µg/gCr	Urinary Creatinine	Oxidative Stress
Zimapán, Hidalgo [29]	90	6–10	186.7 µg/L	19.9 µg/L	28.5 µg/L	135.7 µg/L	None	Immunosu-ppression
Villa de la Paz and Morales, San Luis Potosí [40]	84	6–12	26.44 µg/gCr				Urinary Creatinine	Epigenetic Imbalance
Villa de Reyes, San Luis Potosí [43]	83	5–12	37.4 µg/L				Urinary Specific Gravity	Early Kidney Damage
Highlands and Centre regions, San Luis Potosí [34]	85	4–11	H: 44.5 µg/gCr M: 16.8 µg/gCr L: 12.8 µg/gCr				Urinary Creatinine	DNA Damage
Yaqui and Mayo Valleys, Sonora [101]	165	6–12	30.9 µg/L				None	Biomonitoring
**Fluoride Biomonitoring**								

**Country/Ref**	**n**	**Age (years)**	**Source of Exposure**	**F Drinking water**	**F in urine**	**Urine Dilution Adj.**	**Health Effect(s)**	

Hermosillo, Sonora [102]	31	8–9	Drinking Water and Food	L: 0.54 mg/L M: 0.78 mg/L H: 2.77 mg/L	L: 0.93 mg/L M: 1.04 mg/L H: 3.1 mg/L	None	Risk Assessment	
L: Moctezuma, San Luis Potosí M: Salitral, San Luis Potosí H: 5 de Febrero, Durango [28]	132	6–10	Drinking Water	L: 0.8 mg/L M: 5.3 mg/L H: 9.4 mg/L	L: 1.8 mg/gCr M: 6.0 mg/gCr H: 5.5 mg/gCr	Urinary Creatinine	Cognitive Deficits	
**Fluoride Biomonitoring**								

**Region/Ref**	**n**	**Age (years)**	**Source of Exposure**	**F Drinking water**	**F in urine**	**Urine Dilution Adj.**	**Health Effect(s)**	

Soledad de Graciano Sánchez, San Luis Potosí [103]	20	6–12	Drinking Water	0.67 mg/L	1.94 mg/gCr	Urinary Creatinine	Increased Apoptosis in PBMC	
Villa de Ramos, San Luis Potosí [104]	72	6–12	Drinking Water	2.3–5.4 mg/L	1.0–8.0 mg/L	Specific Gravity	Inflammatory Expression Genes	
Salinas de Hidalgo, San Luis Potosí [105]	111	6–12	Driking Water	4.54 mg/L	3.14 ± 1.09 mg/L	Specific Gravity	Dental Fluorosis Prevalence (95%)	
Villa de Reyes, San Luis Potosí [43]	83	5–12	Drinking Water	2.47 mg/L	50th: 2.18 mg/L	None	None	

*Abbreviations:* SumAs, sum of arsenic; H, high; M, medium; L, low; NR, non-reported; PBMC, peripheral blood mononuclear cells. *Notes:* Arsenic levels were quantified in urine samples. SumAs: As(III) + As(V) + MAs+ DMAs.

Urinary fluoride BE values for children have been reported [84]. Based on the guidance value reported by USEPA (RfD = 0.08 mg/Kg-day), a BE value of 1.0 mg/L for children ages 3 to 6 and 1.2 mg/L for children ages 6 to 10 have been proposed. Biomonitoring data of urinary fluoride in children from nonendemic fluoride regions have been reported in some countries (Table 3). The mean levels and 50th percentiles of urinary fluoride concentration reported by those studies are below BE value for fluoride (1.2 mg/L), including Mexico. However, 95th and 75th percentile values reported from Canada and China present urinary fluoride levels above BE value. In general, low fluoride concentrations in water (<1.5 mg/L) were reported for these nonendemic fluoride regions; however, water fluoridation programs have been applied for Canada and the United Kingdom, and alternative sources of fluoride exposure could have also occurred.

Table 4 shows biomonitoring data of urinary fluoride in Mexican children from endemic fluorosis regions. However, most of these studies have been performed to investigate fluoride toxicity. In general, most urinary fluoride concentrations reported are above the BE value for fluoride (1.2 mg/L), even in regions with low fluoride concentration in the water. In Mexico, alternative sources of fluoride exposure, such as salt, toothpaste, soil, and food, can also occur. However, it is common to consider only one or two sources, which leads to the potential underestimation of fluoride exposure.

## Discussion

According to the information gathered, a conservative estimation put approximately 6.5 million children in health risk due to arsenic and fluoride exposure in Mexico. These child populations are at an increased risk for impaired neuronal development that might lead to lower learning abilities, higher susceptibility to infectious diseases and inflammation, and chronic conditions that are associated with cancer and degenerative diseases in adulthood.

Thus, using BE for urine samples constitutes a valuable tool fpr identifying child populations at risk. This is a relatively noninvasive methodology to identify exposure that could be implemented in the identified endemic regions. Also, if international regulatory measures are to be considered, operative guidelines should be established to make studies comparable on a global scale.

### Arsenic and Fluoride BE Values for Mexican Population

Urine is the more reliable biospecimen to assess arsenic and fluoride exposure. However, the values need to be adjusted for dilution due to the variable hydration status of the participants. Although adjustment by urinary creatinine is traditionally used, this could result in biased estimates because the urinary biomarker of exposure and creatinine excretion may be affected differentially due to physiological and pathophysiological conditions [106]. Urine specific gravity may be a more appropriate method for urine dilution adjustment in human biomonitoring studies.

The implementation of a global urinary RV for arsenic is challenging. BE and RV_95_ are regional, well-established RV values for U.S. and German populations. Currently, there are no unexposed background RV or guidance values of arsenic in urine for the Mexican population. Arsenic levels in nonendemic regions, like Yucatan, Mexico, are below 10 μg/L and can be an approximation of how the RV could be for the Mexican population [87,88]. The BE of 6.4 μg/L proposed by Hays et al. cannot be realistically adopted by Mexico [78]. Hence, we propose adopting the RV_95_ of 15 μg/L proposed by Schultz et al [107]. Although the RV_95_ represents the basal levels of urine arsenic for a specific country (Germany), it is an achievable RV, especially if the Mexican guideline for arsenic in drinking water is reduced to 10 μg/L. Finally, this tool could be used for large and continuous national biomonitoring studies to establish basal RV.

Limited worldwide biomonitoring data for fluoride was available. Aylward et al. proposed a BE value for urinary fluoride that considers fluoride excretion patterns in children and that can be used to assess fluoride exposure in child populations [84]. It is important to mention that this BE value is based on severe dental fluorosis as a disease criterion derived from a non-Mexican child population (Table 2). In Mexico, high dental fluorosis prevalence rates have been reported even in regions with fluoride water concentrations <1.5 mg/L, suggesting alternative sources of fluoride exposure [57,71,97]. Also, dose-response association between fluoride intake and dental fluorosis suggests that the critical limit in guidance values may not be safe for Mexican children and should be revised in further research to establish recommendations for fluoride distribution schemes and regulations in water, salt, food, and toothpaste in Mexico [96]. Many endemic-fluorosis regions in Mexico have been identified, and the mean urinary fluoride concentrations in some of them are around 2 to 3 mg/L (Table 4). However, no urinary RV or available data from nonendemic regions are reported, and most of the studies performed in Mexico that evaluate fluoride toxicity do not report urine fluoride concentrations. Considering the limited information in Mexico and the limited biomonitoring data from other nonendemic populations, we propose that a BE value of 1.2 mg/L be adopted as a reference to assess fluoride exposure in Mexican children. Nevertheless, as in the case of arsenic, future biomonitoring studies performed in Mexican populations are necessary to establish RV; furthermore, other nonskeletal diseases should be considered for BE derivation.

Although a majority of BE calculations are made assuming there are no differences between child and adult populations, considering cancer as the maximum adverse consequence to chronic exposure, this may not be the best option for the child population. Not all environmental insults lead to cancer; however, exposure to insults may create equally incapacitating health risks. Therefore, it is necessary to reconsider other noncommunicable diseases (NCD) in the derivation of BE, such as those related to neurotoxicity and immunotoxicity that are reported in various epidemiological studies in children (Table 4) [108].

### Cost of Exposure to Arsenic and Fluoride

Nearly two-thirds of deaths caused by environmental risk factors are due to NCD, such as obesity, diabetes, cardiovascular disease, chronic respiratory diseases, neurological diseases, and cancer [109,110]. Both arsenic and fluoride exposure have been linked to NCD [111,112]. NCD are considered a major risk to economic loss, and low-income and middle-income economies are highly vulnerable. In Argentina, Brazil, Colombia, and Mexico, the cumulative loss of gross domestic product from heart disease, stroke, and diabetes between 2006 and 2015 was $13.54 billion [113]. Thus, prevention of arsenic- and fluoride-related diseases, by minimizing exposure of highly vulnerable populations such as children and pregnant women, appears to be a reasonable strategy to reduce the high demand and cost in health services in the long term. For example, Nigra et al. assessed the health benefits of the U.S. reduction of arsenic in drinking water from 50 μg/L to 10 μg/L since 2006 [114]. Over a 10-year period, the levels of DMAs fell by 17%, and a reduction of 200 to 900 lung and bladder cancer cases per year was estimated, if this reduction remains across a lifetime, which also reduces health services costs. Also, in a cross-sectional study performed in Chinese children (n = 26,931), a significant dental fluorosis rate reduction was reported after 10 years of safe fluoride drinking water supply [97].

However, in the case of fluoride, it has been found in recent years that many populations across the world where fluoride exceeds the permitted levels, dental fluorosis has been observed. Moreover, this condition is now linked to other NCD, such as neurological or endocrine illness [51]. Ko and Thiessen estimated that in severe fluorosis children’s teeth need porcelain veneer treatments. If they are replaced every 12 years, the lifetime cost of veneers for a child with moderate or severe fluorosis would be at least $4,434 [115].

Finally, disease estimation gives us another insight into the impact of exposure to arsenic and fluoride. For arsenic, cancers and skin lesions are commonly used as indicators. Worldwide, the burden of disease due to skin lesions caused by arsenic in drinking water ranges from 1.5 to 6.7 disability adjusted life years (DALYs) per 1,000 population [116]. Each year, 9,129 to 119,176 additional cases of bladder cancer; 11,844 to 121,442 cases of lung cancer; and 10,729 to 110,015 cases of skin cancer worldwide are attributable to inorganic arsenic in food [117]. For fluoride, the global burden of disease based on the exposure-response relationship for skeletal fluorosis ranges from less than 1 to 20 DALYs per 1,000 population [118].

## Final remarks

Given the potential adverse health effects related to arsenic and fluoride, immediate measures should be taken to reduce exposure, particularly for vulnerable populations and specifically for children and pregnant women.

The BE values presented here are proposed as a starting point for regulatory purposes; however, it is emphasized that these BE values will have to be adjusted as more information becomes available about individual exposure and co-exposure to arsenic and fluoride.
